# Six-year monitoring of pesticide resistance in the Colorado potato beetle (*Leptinotarsa decemlineata* Say) during a neonicotinoid restriction period

**DOI:** 10.1371/journal.pone.0303238

**Published:** 2024-05-06

**Authors:** Frantisek Kocourek, Petr Dolezal, Ervin Hausvater, Tereza Horska, Bruno Sopko, Petr Sedlak, Vladimira Sedlakova, Jitka Stara

**Affiliations:** 1 Crop Research Institute, Drnovska 507/73, Ruzyne, Czechia; 2 Department of Protection, Potato Research Institute Havlickuv Brod, Ltd., Havlickuv Brod, Czechia; 3 Faculty of Agrobiology, Department of Genetics and Breeding, Czech University of Life Sciences Prague, Food and Natural Resources, Suchdol, Czechia; University of Leipzig Faculty of Life Sciences: Universitat Leipzig Fakultat fur Lebenswissenschaften, GERMANY

## Abstract

The Colorado potato beetle (CPB; *Leptinotarsa decemlineata*) is an important potato pest with known resistance to pyrethroids and organophosphates in Czechia. Decreased efficacy of neonicotinoids has been observed in last decade. After the restriction of using chlorpyrifos, thiacloprid and thiamethoxam by EU regulation, growers seek for information about the resistance of CPB to used insecticides and recommended antiresistant strategies. The development of CPB resistance to selected insecticides was evaluated in bioassays in 69 local populations from Czechia in 2017–2022 and in 2007–2022 in small plot experiments in Zabcice in South Moravia. The mortality in each subpopulation in the bioassays was evaluated at the field-recommended rates of insecticides to estimate the 50% and 90% lethal concentrations (LC_50_ and LC_90_, respectively). High levels of CPB resistance to *lambda*-cyhalothrin and chlorpyrifos were demonstrated throughout Czechia, without significant changes between years and regions. The average mortality after application of the field-recommended rate of *lambda*-cyhalothrin was influenced by temperature before larvae were sampled for bioassays and decreased with increasing temperature in June. Downwards trends in the LC_90_ values of chlorpyrifos and the average mortality after application of the field-recommended rate of acetamiprid in the bioassay were recorded over a 6-year period. The baseline LC_50_ value (with 95% confidence limit) of 0.04 mg/L of chlorantraniliprole was established for Czech populations of CPBs for the purpose of resistance monitoring in the next years. Widespread resistance to pyrethroids, organophosphates and neonicotinoids was demonstrated, and changes in anti-resistant strategies to control CPBs were discussed.

## Introduction

The Colorado potato beetle (CPB), *Leptinotarsa decemlineata* (Say), is the most serious pest afflicting potatoes in North America and many areas of Europe. Consumption by larvae and adults can cause severe defoliation of potato plants and lead to yield losses. Due to the intensive use of chemical insecticides and the adaptability of CPBs, a wide range of insecticides have failed to control CPBs because of the development of resistance. CPBs have an extraordinary ability to evolve resistance to all insecticidal chemistries [1]. The first case of CPB resistance to insecticides occurred in 1952 for DDT [2]. In 1958, resistance to dieldrin was reported, followed by resistance to other chlorinated hydrocarbon chlordane and organophosphate diazinon [3]. In Czechia, the resistance of CPBs to DDT was reported in 1968 [4]. Since the middle of the last century, CPBs have developed resistance to over 55 different insecticides, including organophosphates [5], pyrethroids [6], neonicotinoids [7] and spinosad [8].

CPB populations resistant to organophosphates and pyrethroids in Czechia were first recorded in 2004 [9]. The first instances of resistance to the neonicotinoid imidacloprid were reported in the USA at the end of the last century [7], only 3 years after its registration. Resistance to neonicotinoids has since spread and expanded to include resistance to thiamethoxam and clothianidin [10–12]. By 2009, 95% of the populations tested from the northeastern and midwestern United States were resistant to imidacloprid [12]. To date, resistance to neonicotinoid insecticides has been reported in China [13], Serbia [14], and Canada [15]. CPB populations resistant to acetamiprid in Czechia were first observed by farmers in 2010, while insecticides based on thiacloprid or thiamethoxam were still efficient (unpublished data). Before the restriction of using chlorpyrifos, thiacloprid and thiamethoxam by EU regulation, these insecticides were the most frequently used insecticides against CPB in Czechia.

All the neonicotinoid insecticides belong to the same mode of action group according to the classification developed by the Insecticide Resistance Action Committee (MOA Group 4A) [16], and their rotation with each other facilitated the development of resistance due to cross-resistance to different neonicotinoid insecticides [10,17]. However, cross-resistance among different chemicals within the same group is often incomplete, with one product performing somewhat better than another [17]. Positive cross-resistance patterns were also demonstrated for neonicotinoids with esfenvalerate and azinphosmethyl [18]. The correlation between CPB mortality when exposed to neonicotinoids and chlorantraniliprole or cyantraniliprole from the group of anthranilic diamide insecticides was usually not significant [17], indicating a low level of cross-resistance. Mota-Sanchez et al. [10] reported low to moderate level of resistance to spinosad of imidacloprid-resistant CPB population from Long Island in 2004 compared to a susceptible CPB laboratory colony. However, Alyokhin et al. [19] reported asymmetric cross-resistance between spinosad and imidacloprid.

The nicotinoid resistance of CPBs is due to overexpression of genes encoding detoxifying enzymes, including cytochrome P450, glutathione S-transferase, UDP-glycosyltransferase, and xenobiotic transporters, such as ATP-binding cassette (ABC) transporters, which participate in the excretion process of metabolized insecticides [15,20]. To date, no mutations have been reported in the alpha subunits of the target site responsible for resistance to neonicotinoids in CPBs, and metabolic resistance is still considered the main mechanism responsible for resistance to neonicotinoids in CPBs.

To date, diamide insecticides seem to be the group of insecticides with preserved efficacy against CPBs worldwide. No resistance to chlorantraniliprole or cross-resistance with other insecticides has been reported in CPBs to date. However, a slight decrease in susceptibility to chlorantraniliprole has been observed in other insects [21,22]. In addition, populations of the diamondback moth *Plutella xylostella* L. (Lepidoptera: Plutellidae) in China were found to be resistant to chlorantraniliprole and cross-resistant to flubendiamide [23]. Therefore, it appears that there is a potential risk of resistance against chlorantraniliprole resulting from cytochrome P450 monooxygenase activity [24]. Currently, when the number of available insecticides is decreasing, there is a need for new possibilities and solutions for CPB control. Direct alternative methods to control CPBs include the use of natural enemies [25,26], plant extracts and botanical insecticides [27] or RNA interference (RNAi) techniques [28].

Recent research on CPBs has shown that polygenic resistance drawn from standing genetic diversity explains genomic patterns of insecticide resistance evolution [29]. CPBs exhibit both population genetic structure [30] and high spatial heterogeneity regarding their level of insecticide resistance [31].

The development of CPB resistance to insecticides has been studied mainly in populations from North America. Although all European populations appear to be descended from a single mtDNA haplotype [32], they can develop resistance to insecticides, raising questions about the mechanisms that contribute to rapid evolution. To date, knowledge about the development of CPB resistance in Europe has been fragmental.

The aims of this study were to i) evaluate the development of CPB resistance to insecticides from pyrethroids, neonicotinoids and organophosphates groups and CPB susceptibility to diamide group in Czechia in the period of 2017–2022 when the registrations of organophosphates and two neonicotinoids were restricted in the EU, ii) compare the resistance of CPBs to the neonicotinoids acetamiprid, thiacloprid and thiamethoxam, iii) evaluate the development of CPB resistance to insecticides from pyrethroids, neonicotinoids and organophosphates groups and CPB susceptibility to diamides in 5 regions of Czechia differing in altitude and climatic conditions, iv) evaluate the efficacy of selected insecticides from pyrethroids, neonicotinoids, diamides, and spinosyns groups and azadirachtin in small plot experiments in the period of 2007–2022, and v) establish the baseline of chlorantraniliprole for CPB from Czechia.

## Materials and methods

### Sampling of insects

Samples of L2 larvae and eggs from 69 selected localities in Czechia, which are situated in the potato-growing regions, were collected from commercial potato fields in 2017–2022. A list of localities, their coordinates and altitudes are provided in S1 Table. According to the latitude, coordinates and type of potato grown, the localities were divided into five regions, i.e., South Moravia, Central Bohemia, Bohemian-Moravian Highlands, Central and North Moravia and South and West Bohemia. The sizes of the areas of collection over 6 years in one locality ranged from 1 to 10 ha. The larvae and eggs were sampled from May to late June before the treatment of the fields with insecticides. The samples for bioassay consisted of at least 400 L2 larvae or 50 egg masses collected in various parts of each field.

The collected CPB larvae and eggs were transported in Styrofoam boxes to the laboratory. The larvae were kept in an insectary at 24°C under a 16/8 h light regime (light/dark) and 60% RH until hatching. Fresh potato foliage of the Magda variety was supplied as food. Magda potato plants (GRIN Czech database, Potato Research Institute, Havlickuv Brod, Czechia) were grown in the greenhouse without any pesticide treatment. The L2 larvae of good fitness were tested for susceptibility to insecticides in the bioassay within 1–2 days after collection.

### Insecticides

Analytical standards of *lambda*-cyhalothrin, acetamiprid, thiamethoxam, chlorpyrifos and chlorantraniliprole were obtained from Sigma‒Aldrich (Czechia). Biscaya 240 OD (240 g thiacloprid per L of solution) was used as the neonicotinoid thiacloprid formulated product, as preliminary trials revealed that technical material is not appropriate (results not shown). The analytical standards of active insecticide ingredients (a.i.) were diluted in acetone (*lambda*-cyhalothrin, acetamiprid, chlorpyrifos and thiamethoxam) or dimethylformamide (chlorantraniliprole) to prepare the desired concentrations for the bioassay. Stock solutions of Biscaya 240 OD were prepared by dissolving 0.4 mL of Biscaya 240 OD formulation (containing 96 mg of thiacloprid) in 2 mL of distilled water and subsequently adjusting the volume to 100 mL with acetone. All further dilutions were made with acetone. In the experiments, the field application doses for *lambda*-cyhalothrin (7.5 g a.i./ha), acetamiprid (12 g a.i./ha), thiamethoxam (20 g a.i./ha), thiacloprid (72 g a.i./ha), chlorantraniliprole (12 g a.i./ha) and chlorpyrifos (300 g a.i./ha) were used, assuming that the highest recommended dose of a commercial product in Czechia was used in 400 L of water per hectare. Three to seven doses of insecticides were used to generate data for calculating dose‒response curves (Tables 1 and S2–S7). Formulated commercial products of chlorantraniliprole, *lambda*-cyhalothrin, acetamiprid, azadirachtin and spinosad dissolved in water were used for the field trials (Table 2).

**Table 1:** The recommended field rates and tested rates of insecticides used in bioassays to evaluate *Leptinotarsa decemlineata* susceptibility from Czechia in 2017–2022, *—application rate of water 400 L/ha.

Tested insecticides	Field rates g a.i./ha	Field rates* (mg a.i./L)	Tested rates (mg a.i./L)
*lambda*-cyhalothrin	7.5	18.8	0.75; 3.75; **18.8**; 93.8; 281; 375; 938
acetamiprid	12	30.0	1.20; 6.00; **30.0**; 60.0; 150
thiacloprid	72	180	7.20; 36.0; **180**; 360; 900
thiamethoxam	20	50.0	2.00; 2.50; 10.0; **50.0**; 100
chlorpyrifos	300	750	7.50; 75.0; 225; **750**; 1,875; 14,648; 146,648
chlorantraniliprole	12	30.0	0.03; 1.20; **30.0**

**Table 2:** The trade names, active ingredients, companies and application rates of pesticides used in the study in Zabcice.

Trade name	Active ingredient	kg; L/ha	Company (permit holder in Czechia)
Coragen 20 SC	chlorantraniliprol	0.06	FMC Agro Ceska republika spol. s r.o.
Karate se Zeon technologii 5 CS	*lambda*-cyhalothrin	0.15	Syngenta Limited
Mospilan 20 SP	acetamiprid	0.06	Nisso Chemical Europe GmbH
NeemAzal T/S	azadirachtin	2.5	Trifolio-M GmbH
SpinTor	spinosad	0.15	Corteva Agriscience Czech s.r.o.

### Topical bioassay

Topical application was used to assess the susceptibility of CPB L2 larvae to selected insecticides from groups of class II pyrethroids (*lambda*-cyhalothrin), organophosphates (chlorpyrifos) and neonicotinoids (acetamiprid, thiamethoxam, thiacloprid). Three replicates of 10 larvae per replicate were treated individually with 1 μL of insecticide solution, which was applied to the dorsal abdominal segment using a Multipette^®^ Plus dispenser (Eppendorf AG, Hamburg, Germany). The control larvae (three replicates of 10 larvae per replicate) were treated with acetone. After treatment, the larvae were placed in plastic cups (Gastro, Czechia, 150 ml volume) containing fresh potato leaves. The cups were sealed with a breathable lid and placed in a climate box at 24°C, 60% RH and 16/8 h light regime (light/dark). The number of severely affected larvae (i.e., dead and moribund) was counted after 24 h.

### Ingestion bioassay

An ingestion bioassay was used to assess susceptibility to the diamide chlorantraniliprole. A leaf dip method (IRAC, Method No: 007) was adopted for the digestion bioassay. Three concentrations of chlorantraniliprole were prepared from serial dilutions with 100 mL of distilled water. Fresh potato leaves were individually dipped into one of the solutions for 5 s, removed, and dried under airflow on filter paper. The treated leaves were then placed in plastic cups (Gastro, Czechia, 150 ml volume), and ten L2 CPB larvae were transferred to each dish. Distilled water was used as a control. All the treatments were replicated three times. After treatment, the cups were placed in a climate box at 24°C, 60% RH and a 16/8 h light regime (light/dark). The number of severely affected larvae (i.e., dead and moribund, i.e. not able to move co-ordinately) was counted after 72 h.

### Field trials

The precise field experiments were carried out in 2007–2022 in Zabcice in South Moravia, a school agricultural company of Mendel University in Brno, located in a new potato growing area. The field plots are planar with elevations of 179–184 m above sea level and belong to the warmest and dry localities in Czechia, with an average annual temperature of 9.2°C and an average total precipitation of 480 mm. These experimental fields serve for long-term experiments and are used exclusively for testing of pesticide efficacy against pest.

The precise experiments were established in a completely randomized design with the control plots included in plots. Common agrotechnics with the application of herbicides and fungicides were used for the cultivation of potatoes. The variants in 4 replicates were established in plots of 25.2–30.0 m^2^. The insecticides and the rates used are presented in Table 2. Formulated commercial products dissolved in water were used for the field applications. Any other insecticides were not applied in the experimental plots. The Rosara cultivar was grown within a row spacing of 30 cm and 75 cm between rows. The pesticides were applied in 400 L of water per ha with a VERMOREL Electric 2000 backpack sprayer. The number of CPB larvae of L1–L4 instars, number of CPB adults, percentage of plant defoliation and numbers of egg masses were evaluated on 10 plants in each replication and variant, depending on the CPB population density. The evaluations were carried out before the insecticide application and subsequently 1–3 days and 7–11 days after the application.

### Data analysis

The bioassay data were analysed by probit analysis using a dose-effect analysis in XLSTAT 2021 (Addinsoft, NY, USA). The doses of insecticides were log10-transformed. The estimates of lethal doses (LC_20_ and LC_50_ for *lambda*-cyhalothrin and LC_50_ and LC_90_ for other insecticides in the study) were fitted with 95% confidence limits. The lethal doses were calculated for each field population. The CPB mortality in each bioassay was corrected using Abbott´s formula. Resistance ratio (RR) calculations were related to the lowest LC_50_ values of acetamiprid, thiacloprid and thiamethoxam that had been recorded for CPB field populations. Resistance ratios were not calculated for *lambda*-cyhalothrin and chlorpyrifos due to the absence of sensitive populations in the bioassays according to the mortality data. The mean mortalities of the field populations exposed to 100% of the recommended dose of insecticides in the bioassays were compared with Kruskal-Wallis test. The efficacy of insecticide application against the CPB larvae of the L1–L4 instars in particular years in the field experiments was evaluated according to [33]. The effects of pesticide, year, climatic region, temperature, and precipitation in June and sum of effective temperature (SET) above 10°C in June on the CPB mortality, LC_50_ and LC_90_ values were evaluated using R statistical software, version 4.3.1 [34]. The weather data from five meteorological stations representing the evaluated regions (i.e. Pohorelice for South Moravia, Doksany for Central Bohemia, Havlickuv Brod for Bohemian-Moravian Highlands, Opava for Central and North Moravia and Klatovy for South and West Bohemia) were obtained from the Czech Hydrometeorological Institute (CHMI, Prague, Czechia). The regions are defined with mean SET above 10°C from 1^st^ January to 30^st^ November in 2017–2022 and mean temperature from 1^st^ January to 30^st^ November in 2017–2022 (S1 Table). To explain the influence of the examined factors, we deployed Bayesian statistics using the BayesFactor package in R [35–37].

## Results

### Development of CPB resistance to *lambda*-cyhalothrin

**Fig 1:**
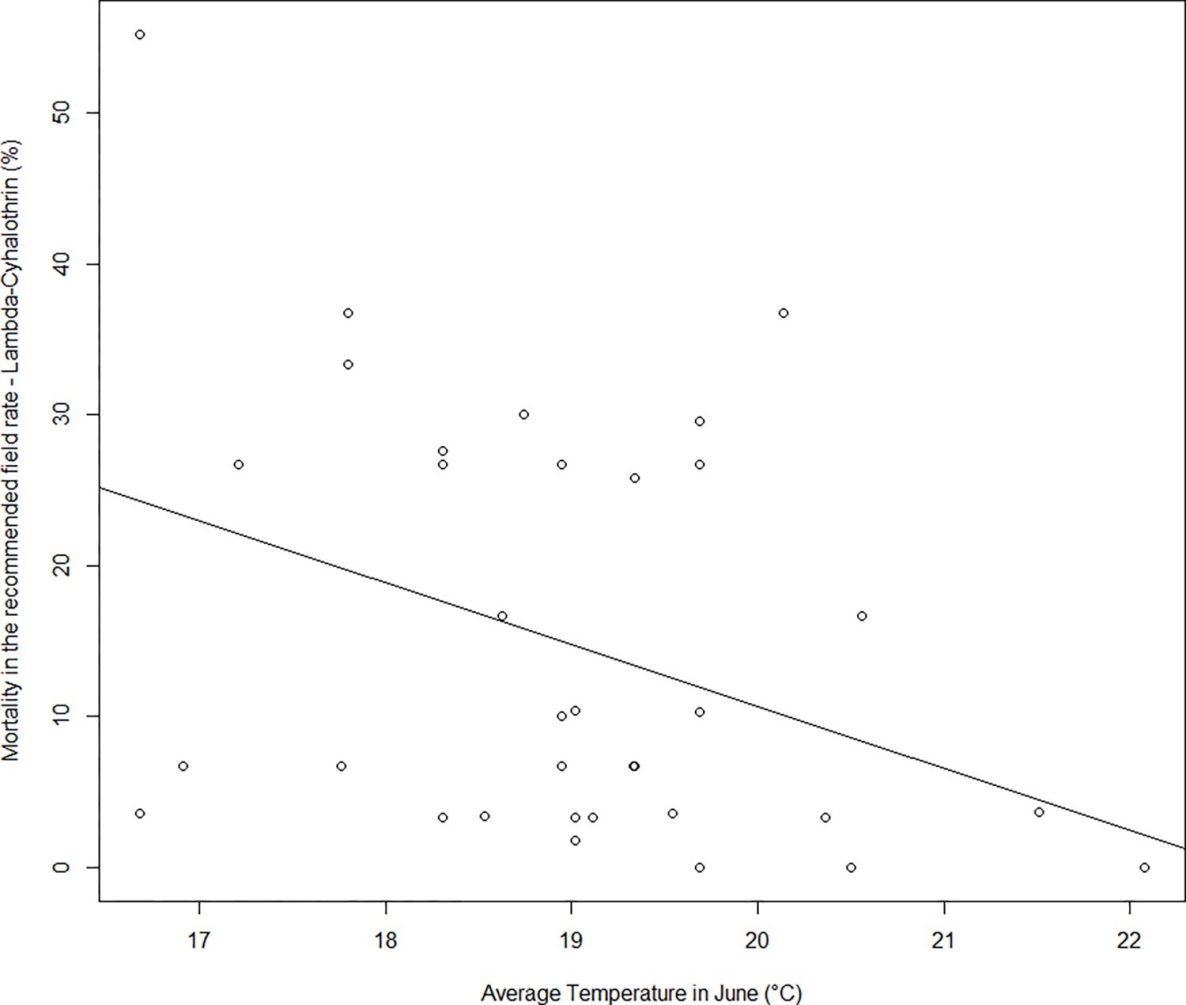
Regression of the mortality of *Leptinotarsa decemlineata* after *lambda*-cyhalothrin application in the recommended field rate (18.75 mg a.i./L) in bioassay and temperature (°C) in June in particular years and regions.

Colorado potato beetle resistance to pyrethroids was monitored in 2017–2022 on 59 local populations of CPBs from Czechia. The results revealed high levels of resistance to *lambda*-cyhalothrin in all the tested CPB populations, with low mortality and high LC_50_ values in all years and regions (Tables 3, 4 and S2). The mortalities in the untreated control variants ranged from 0–10.0%. The mortalities of CPB larvae after application of *lambda*-cyhalothrin at the recommended field rate ranged between populations from 0% to 55.2% in the 2017–2022 period (S2 Table). The mortalities after application of *lambda*-cyhalothrin at a rate of 281 mg/L (15 times the recommended field rate) ranged from 3.33% to 86.7% (S2 Table). The low mortality observed at the highest rate of *lambda*-cyhalothrin in most of the tested CPB populations did not enable us to determine the LC_90_ values. Hence, the LC_20_ values were determined (S2 Table). Kruskal–Wallis analysis found differences in the levels of resistance to *lambda*-cyhalothrin according to mortality at the field recommended rate in particular years of evaluation (Kruskal–Wallis: H = 12.23, α = 0.05, p = 0.030). No difference was found by Kruskal–Wallis analysis in the level of resistance to *lambda*-cyhalothrin between the regions of CPB sample origin (Kruskal–Wallis: H = 2.01, α = 0.05, p = 0.717). The median LC_50_ values were 1,765 mg/L in 2017 and 862.5 mg/L in 2022. The most sensitive population of CPBs to *lambda*-cyhalothrin was Procevily in 2020, with an LC_20_ value of 2.98 mg/L, and the most resistant population was Nemcovice in 2022, with an LC_20_ value of 146,998 mg/L. In most of the populations, very low mortality after application of *lambda*-cyhalothrin at the recommended field rate was observed. According to the mortality data, no population sensitive to *lambda*-cyhalothrin was recorded in the 2017–2022 period. The statistical analysis shown a significant negative relationship between the level of CPB resistance to *lambda*-cyhalothrin, expressed as mortality at the recommended field rate and the average temperature in June in a given year and region (mortality = (−4.113 ± 1.842)*temperature-June + (92.919 ± 35.098), p = 0.033, R = -0.36, Fig 1). Larvae of CPB populations developing at higher temperatures in the field had higher resistance to pyrethroids in the bioassays.

**Table 3:** Mean *Leptinotarsa decemlineata* mortality (%) at 100% of the field-recommended rate (mean from all samples) in particular years. Standard deviation and maximum mortality from all of the samples are given in brackets. x—no data available.

Year	*lambda*-cyhalothrin	acetamiprid	thiacloprid	thiamethoxam	chlorpyrifos	chlorantraniliprole
2017	7.5 (10.7, 30.0)	36.8 (26.4, 86.7)	47.3 (27.1, 93.3)	93.3 (5.8, 100)	22.1 (16.2, 46.7)	x
2018	22.0 (13.4, 36.7)	47.7 (19.1, 73.3)	76.7 (17.3, 100)	91.5 (8.7, 100)	27.5 (21.4, 56.7)	x
2019	4.4 (6.6, 16.7)	54.6 (30.2, 96.7)	75.5 (28.2, 100)	x	25.7 (24.6, 64.3)	x
2020	16.5 (17.7, 55.2)	39.5 (6.9, 46.7)	x	x	36.2 (24.3, 60.0)	92.2 (5.08, 96.7)
2021	5.3 (2.6, 10.4)	50.1 (35.0, 100)	x	x	x	96.9 (3.46, 100)
2022	10.8 (13.1, 40.0)	60.7 (28.0, 93.3)	x	x	x	100 (0, 100)

**Table 4:** Mean *Leptinotarsa decemlineata* mortality (%) at 100% of the field-recommended rate (mean from all samples) in particular regions of Czechia. Standard deviation and maximum mortality from all of the samples are given in brackets. x—no data available, nd–standard deviation not defined (only one population tested in region).

Region	*lambda*-cyhalothrin	acetamiprid	thiacloprid	thiamethoxam	chlorpyrifos	chlorantraniliprole
South Moravia	16.7 (19.5, 6.7)	31.0 (16.7, 46.7)	71.4 (27.4, 100)	x	x	100 (nd, 100)
Central Bohemia	8.40 (10.6, 40.0)	51.1 (25.2, 89.7)	64.8 (31.4, 100)	95.7 (6.20, 100)	26.5 (20.5, 64.3)	97.4 (4.30, 100)
Bohemian-Moravian Highlands	9.10 (8.30, 25.8)	65.3 (35.3, 93.3)	78.9 (13.4, 93.3)	x	21.1 (23.7, 46.7)	100 (nd, 100)
Central and North Moravia	15.7 (12.4, 7.6)	59.9 (23.3, 100)	65.8 (5.70, 73.3)	86.9 (8.80, 96.8)	37.6 (21.0, 56.7)	93.1 (nd, 93.1)
South and West Bohemia	14.4 (16.2, 55.2)	45.0 (28.2, 96.7)	70.3 (28.8, 100)	88.2 (9.00, 100)	22.9 (22.9, 55.0)	97.8 (3.87, 100)

### Development of CPB resistance to chlorpyrifos

**Fig 2:**
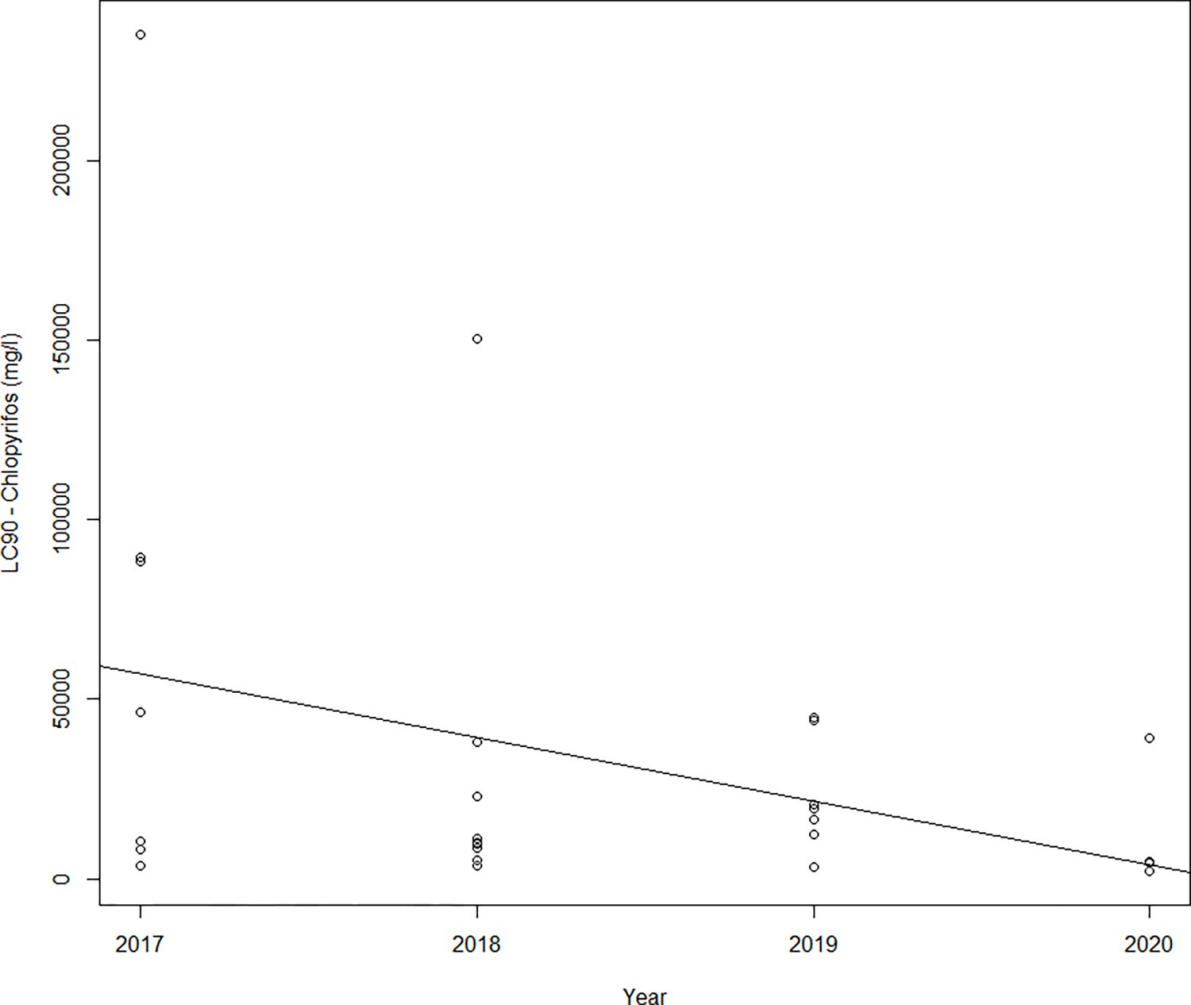
Regression of the LC_90_ values of chlorpyrifos and year of bioassay.

Colorado potato beetle resistance to chlorpyrifos was monitored in 2017–2020 in 31 local populations from Czechia. The mortalities in the recommended application rate ranged from 0 to 64.3% in the 2017–2020 period (S3 Table). Kruskal–Wallis analysis did not find differences in the level of resistance to chlorpyrifos according to mortality at the field recommended rate in particular years of evaluation (Kruskal–Wallis: H = 12.28, α = 0.05, p = 0.734) and regions of CPB sample origin (Kruskal–Wallis: H = 2.05, α = 0.05, p = 0.562). The median LC_50_ values were 1,681.3 mg/L in 2017 and 1,042.5 mg/L in 2020. The lowest LC_50_ value of chlorpyrifos, 397.5 mg/L, was obtained for Utechovicky u Pelhrimova population in 2017. The highest LC_50_ value of 32,762 mg/L was obtained for Pracejovice population in 2020. In most of the populations, very low mortality after the application of chlorpyrifos at the recommended field rate was observed. According to the mortality data, no population sensitive to chlorpyrifos was recorded in the 2017–2020 period. The LC_90_ values of chlorpyrifos significantly decreased in the 2017–2020 period (LC_90_ = (−17,739 ± 8,790)*year + 35,836,988 ± 17,740,590), p = 0.054, R = −0.36, Fig 2).

### Development of CPB resistance to neonicotinoids

**Fig 3:**
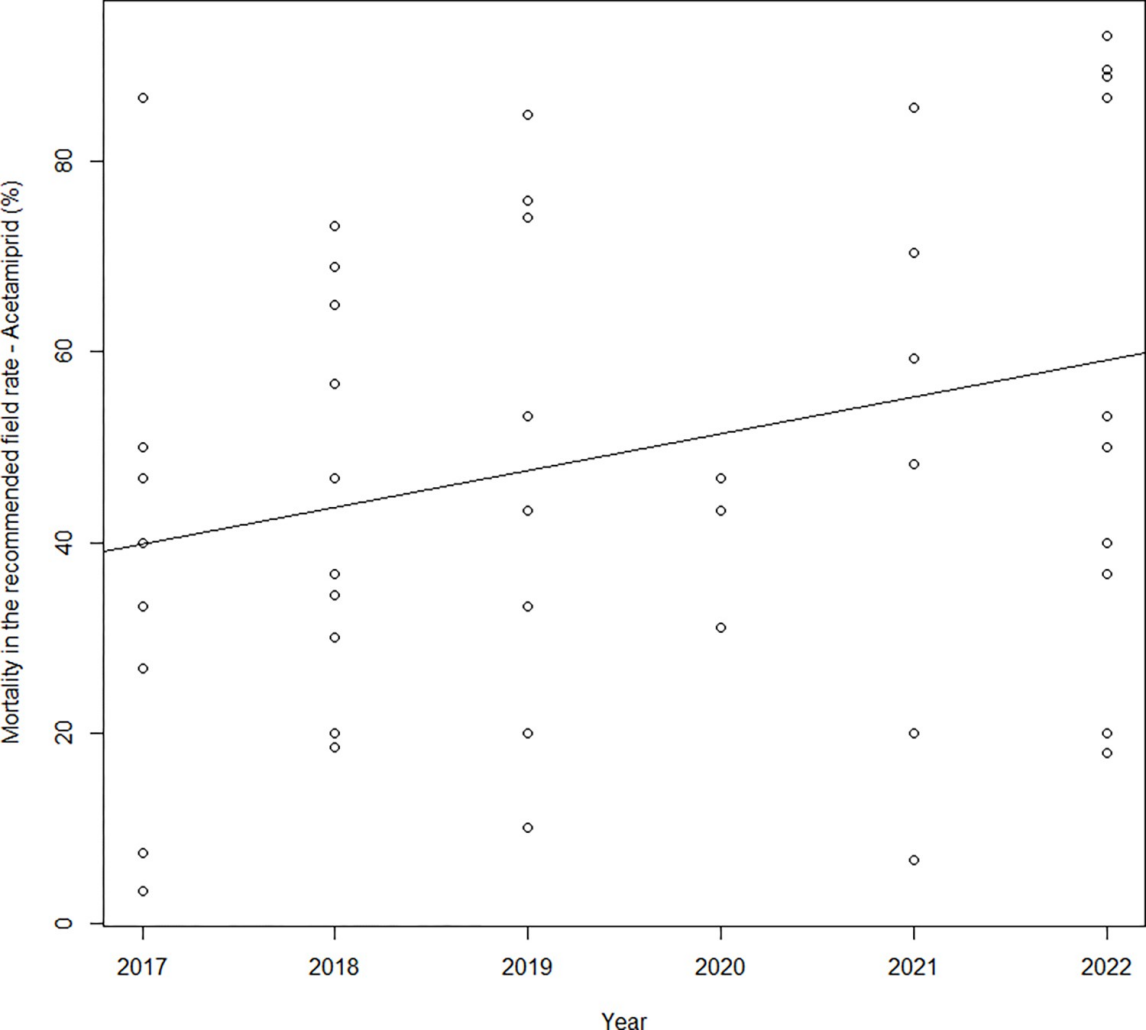
Regression of the mortality of *Leptinotarsa decemlineata* after acetamiprid application in the recommended field rate and year of bioassay.

Colorado potato beetle resistance to acetamiprid was monitored in the 2017–2022 period in bioassays on 56 local populations from Czechia. The mortalities after application of acetamiprid at the recommended field rate ranged from 3.3% to 100% (S4 Table). A statistically significant increase in mortality from 2017–2022 was shown after the application of acetamiprid at the recommended field rate (mortality = (3.866 ± 1.897)*year + (-7,758 ± 3,831), p = 0.048, R = 0.28, Fig 3).

Kruskal–Wallis analysis did not find differences in the level of resistance to acetamiprid according to mortality at the field recommended rate in particular years of evaluation (Kruskal–Wallis: H = 2.08, α = 0.05, p = 0.353) and regions of CPB sample origin (Kruskal–Wallis: H = 5.76, α = 0.05, p = 0.217). The median LC_50_ values were 39.0 mg/L and 22.3 mg/L in 2017 and 2022, respectively. The lowest LC_50_ value of 0.53 mg/L was obtained for Chotikov population in 2022. The highest LC_50_ value of 1,667.3 mg/L was obtained for Ruzyne population in 2021. The resistance ratio (RR) between these two populations was 3,132, whereas RR values below 20 were calculated for 6 local populations from the 56 evaluated populations (Table 5). High mortality at the recommended field rate of acetamiprid in these populations corresponded with a low RR. Mortalities after application of acetamiprid in the field-recommended rate higher than 80% were observed in 11 populations from the 50 evaluated, when most of these populations were evaluated in 2021 and 2022. This indication of slow recovery of sensitivity to acetamiprid can be explained by the absence of selection pressure of the restricted neonicotinoids thiacloprid and thiamethoxam.

**Table 5:** Resistance ratios for acetamiprid, thiacloprid and thiamethoxam related to the lowest LC_50_ values recorded in *Leptinotarsa decemlineata* collections from 2017 to 2022, x–no data available, nd—fit LC with unreal data (i.e.> 999,999 mg/L).

Year	Population	acetamiprid	thiacloprid	thiamethoxam
2017	Dolanky nad Ohri	596	11.2	28.6
	Ruzyne	59.2	3.00	9.10
	Semice	195	19.7	x
	Tesovice	79.8	4.60	x
	Travcice	66.7	10.4	7.00
	Troubsko	364	11.7	x
	Utechovicky u Pelhrimova	x	2.00	x
	Vilemov	21.0	3.40	x
	Vysoka u Pribrame	54.5	9.10	x
2018	Bozice	x	1.90	x
	Celakovice	292	1.90	1.00
	Dolni Zivotice	69.0	2.80	5.60
	Drachkov	140	6.40	13.2
	Javornik	66.2	4.00	13.8
	Obristvi	31.6	3.60	2.30
	Ostretice	159	6.40	19.0
	Popovice	25.9	x	x
	Procevily	35.8	1.00	5.10
	Prerov nad Labem	25.1	2.37	8.70
	Ruzyne	150	3.70	12.0
	Semice	x	1.30	x
	Stryckovice	127	4.40	12.9
	Travcice	42.0	1.50	9.50
	Vicov	78.4	3.40	10.8
2019	Hrdly	34.4	x	x
	Libocany	42.3	13.1	x
	Procevily	37.8	3.10	x
	Ruzyne	19.2	2.70	x
	Stankov	109	2.00	x
	Troubsko	127	3.30	x
	Vrsovice	138	2.70	x
	Zalezlice	113	2.00	x
	Valecov	109	1.40	x
2020	Obristvi	55.4	x	x
	Pracejovice	96.2	x	x
	Ruzyne	51.2	x	x
	Travcice	1,338	x	x
2021	Frycovice	99.5	x	x
	Nemcovice	202	x	x
	Obristvi	18.0	x	x
	Procevily	177	x	x
	Ruzyne	3,132	x	x
	Semice	20.4	x	x
	Travcice	33.1	x	x
	Vrsovice	nd	x	x
2022	Bezdekov pod Tremsinem	70.0	x	x
	Chotikov	1.00	x	x
	Drachkov	23.5	x	x
	Holany	76.5	x	x
	Nemcovice	325	x	x
	Obristvi	13.7	x	x
	Podsedice	59.2	x	x
	Procevily	9.40	x	x
	Ruzyne	257	x	x
	Semice	24.7	x	x
	Svitavy	4.10	x	x
	Travcice	255	x	x
	Zahori u Milicina	20.0	x	x
	Zabcice	59.6	x	x

Colorado potato beetle resistance to thiacloprid was monitored from 2017–2019 in bioassays on 33 local populations from Czechia. The mortalities after application of thiacloprid at the recommended field rate ranged from 11.1% to 100% (S5 Table). The lowest and the highest LC_50_ values of 26.25 mg/L and 517.5 mg/L were obtained from Procevily population in 2019 Semice population in 2017, respectively. The resistance ratio between these two populations was 19.7; however, RR values below 10 were observed in most populations, except for 5. High mortality after application of thiacloprid in the field recommended rate in 5 out of 9 evaluated populations in 2019 did not indicate any trend in decrease of CPB sensitivity to thiacloprid in the period of 2017–2019.

The resistance of CPBs to thiamethoxam was evaluated in 2017–2018 in 15 populations. The mortalities of CPBs after application of thiamethoxam in the field were high and ranged from 79.3% to 100%. The resistance ratios were estimated based on the population Obristvi in 2018 with the lowest LC_50_ value of 2.6 mg/L. The RR values ranged from 2.3 to 28.6, and in most of the populations, the RR corresponded well with the high mortality at the recommended field rate (Tables 5 and S6).

Kruskal–Wallis analysis showed significant differences in mortality at the field recommended rates of three different neonicotinoids (Kruskal–Wallis: H = 24.93, α = 0.05, p < 0.0001).

### Efficacy of chlorantraniliprole against resistant populations of CPB

The efficacy of chlorantraniliprole was monitored in the 2020–2022 period in bioassays on 17 local populations from Czechia. The mortalities after application of chlorantraniliprole at the recommended field rate were high and ranged from 86.7 to 100% (S7 Table). Any decrease in the sensitivity of CPBs to chlorantraniliprole was observed in the period of 2020–2022. The LC_50_ value of 0.04 mg/L for chlorantraniliprole was established as the baseline for Czech populations of CPBs. This LC_50_ value was obtained in four populations from Czechia in 2021 and 2022 as the lowest LC_50_ from the populations tested (S7 Table).

### Efficacy of insecticides against CPBs in small plot experiments

The average efficacies of five insecticides on CPB larvae in small plot experiments in the Zabcice locality from 2007–2022 are presented in Table 6. The insecticides are sorted from least efficient to most efficient. *Lambda*-cyhalothrin had very low efficacy (below 50% on average). This result corresponds well with the results of monitoring the resistance in the bioassays. The efficacies of *lambda*-cyhalothrin in the period from 2018–2022 ranged between 0 and 20%. In comparison to the other evaluated insecticides, the efficacy of *lambda*-cyhalothrin 7 days after application was significantly lower (by 21% on average) than the efficacy 2 days after application. Most likely, some of the larvae dropped from the plants after the treatment, recovered and returned to the plants by 7 days after the treatment.

**Table 6:** Efficacy (% mortality) of insecticides against CPB larvae evaluated 2 and 7 days after the treatment in small plot experiments in Zabcice during 2007–2022, nd = data not available.

Year	Evaluation	azadirachtin	acetamiprid	spinosad	chlorantraniliprole	*lambda*-cyhalothrin
2007	2 days	nd	86.5	nd	nd	33.2
2007	7 days	nd	72.7	nd	nd	17.7
2009	2 days	53.7	69.4	84.6	nd	58.3
2009	7 days	59.2	41.4	84.8	nd	57.6
2010	2 days	2.2	98.8	94.2	nd	nd
2010	7 days	84.4	90.2	100	nd	nd
2011	2 days	32.3	88.4	99.8	nd	nd
2011	7 days	58.8	95.5	100	nd	nd
2012	2 days	87.6	97.4	100	nd	nd
2012	7 days	94.3	85.2	96.6	nd	nd
2013	2 days	35.6	79.5	98.3	59.5	nd
2013	7 days	8.85	84.4	99.5	91.1	nd
2014	2 days	15.6	80.8	99.5	72.7	nd
2014	7 days	85.8	87.2	100	97.5	nd
2015	2 days	19.5	82.1	99.3	nd	57.4
2015	7 days	65.0	82.0	99.7	nd	77.8
2017	2 days	55.7	81.4	89.4	73.8	nd
2017	7 days	51.5	71.3	99.7	98.7	nd
2018	2 days	24.5	83.2	92.8	67.8	41.7
2018	7 days	52.4	81.2	100	97.5	0
2019	2 days	56.3	57.7	95.1	81.2	37.1
2019	7 days	91.3	79.1	98.8	98.8	21.5
2020	2 days	34.8	46.0	94.9	86.4	47.5
2020	7 days	39.7	0	95.2	100	1.14
2021	2 days	41.3	65.9	97.2	83.3	48.4
2021	7 days	71.7	53.5	98.8	97.3	0
2022	2 days	53.8	68.7	100	82.9	41.6
2022	7 days	86.6	59.1	100	96.1	22.9

The efficacies of azadirachtin were variable and ranged from 8.85% to 94.3% without any trend between years of evaluation. The average efficacy of azadirachtin 7 days after the treatment reached 61%. The efficacy after 7 days from the treatment was higher than after 2 days from the treatment. Accordingly, the effect of azadirachtin on the CPB larvae was slow, and mortality gradually increased as a result of the treatment. The efficacy of azadirachtin, in comparison to the other insecticides (except *lambda*-cyhalothrin), was low and without any indication of the development of resistance.

The efficacy of acetamiprid decreased during the period of evaluation from 90.2% in 2010 to 59.1% in 2022 (Kruskal–Wallis: H = 28.5, α = 0.05, p = 0.008). This trend is evidence of the development of resistance to acetamiprid in one population of CPBs during the 14-year period. No difference in efficacy was observed when it was evaluated 2 and 7 days after the treatment. Therefore, the efficacy of acetamiprid in 2022 was insufficient from a practical point of view.

High efficacy without any decreasing trend was observed for chlorantraniliprole. The efficacy 2 days after the treatment was 76% on average and increased to 97% on average by the 7^th^ day after the treatment. Having a slow effect on the CPB larvae, the chlorantraniliprole efficacy consequently increased. These results provide important information, as they advise potato growers to evaluate insecticide efficacy not before 7 days after treatment.

The efficacies of spinosad were high and ranged between 84.8 and 100% without any incidence of a decrease in efficacy. No differences in efficacy were found 2 and 7 days after the treatment. The effect of spinosad on the CPB larvae was fast and caused very rapid mortality after treatment.

## Discussion

### Background of the development of CPB resistance in Czechia

Potatoes are grown in Czechia on an area of approximately 20,000 ha [38]. The need for CPB control in potatoes has increased mainly in new potato areas (Central Bohemia and South Moravia regions). Great changes in the spectrum of insecticides registered for CPB control in potatoes have occurred in recent years. The use of wide-spectrum organophosphates, carbamates and most of neonicotinoids was consequently restricted [39–41]. The number of new insecticides registered is limited, and their use requires more frequent and precise monitoring of pest incidence in the field. In addition to changes in the spectra of available insecticides, the high speed of selection of CPB resistance and changes in weather conditions as a consequence of global warming contribute to the increased need for CPB control in potatoes. In connection with weather changes, CPBs produce two generations per year not only in warm areas but also in higher locations throughout Czechia. In contrast, the second generation of CPBs can be absent in warm areas due to a lack of precipitation. However, the main reason for the trouble with CPB control in Czechia is the rapid selection of resistance to insecticides.

### Speed and stability of the development of CPB resistance

The evaluation of CPB resistance in 2017–2022 presented in this paper did not find any population of CPBs sensitive to organophosphates and pyrethroids in Czechia (Table 3). The resistance of CPBs to organophosphates was selected in Czechia as a result of the selection pressure of pesticides used for CPB control in potato fields; no resistance to organophosphates was recorded in the populations introduced to Czechia. In the 1950s, CPB resistance to DDT was shown in Czechia [4]. As the mechanism of resistance to DDT is the same as that of resistance to pyrethroids, local populations of CPBs in Czechia have the same history of resistance selection to pyrethroids from the 1950s to 2022. According to Brevik et al. [42], the development of CPB resistance to insecticides is very fast. For example, resistance to DDT was reported after 22 and 375 generations in CPBs and *Myzus persicae*, respectively, while the mean duration between the introduction of an insecticide and the first report of resistance in pests was 66 generations [42].

Our results showed that CPB resistance to insecticides remains in the population for a long time in the absence of any selection pressure from insecticides. The decrease in the LC_90_ value of chlorpyrifos in the 2017–2020 period indicates a recovery of CPB sensitivity to chlorpyrifos (S3 Table). Chlorpyrifos was not used for CPB control in the field in the evaluated period from 2017–2020, and the recovery of sensitivity to chlorpyrifos without selection pressure was very slow.

The CPB exhibits both population and genetic structure and high spatial heterogeneity in levels of insecticide resistance [17,30]. Temporal and spatial samples of beetles from the same field population or nearby fields can have very different sensitivities to the same insecticide [43,44]. Variations in sensitivity to insecticides, mainly azadirachtin, *lambda*-cyhalothrin and acetamiprid, were also observed in our small plot experiments in one locality during the 2007–2022 period (Table 6). According to Chen et al. [29], polygenic resistance drawn from standing genetic diversity in CPBs explains the genomic patterns of insecticide resistance evolution.

### Impact of environmental stress on resistance levels

The variability in susceptibility to novel insecticides among populations appears to be due to differences in the expression of detoxification mechanisms and cuticular proteins [45]. These patterns suggest that other sources of environmental stress associated with climatic conditions at the regional scale may have influenced CPB resistance [29]. Our results indicate that the mortality of CPB larvae after pyrethroid application in the bioassay decreases with increasing temperature of the environment in June (Fig 1), i.e., in the period of development of eggs and larvae before the sampling for the bioassay. Accordingly, larvae of CPB populations developing at higher temperatures in the field had higher resistance to pyrethroids in the bioassays. Such an impact of environmental stress on the performance and survival of CPBs following insecticide exposure and tolerance to insect pathogens was reported previously by Kryukov et al. [46], where CPBs preexposed to heat stress showed reduced tolerance to *Beauveria bassiana* and insecticidal proteins from *Bacillus thuringiensis*.

### Development of CPB resistance to neonicotinoids

Development of resistance to neonicotinoids in Czechia was documented in the bioassays in 2017–2022 (Table 3) as well as in the small plot experiments in 2007–2022 (Table 6). We found differences in the development of resistance to various neonicotinoids, i.e., acetamiprid, thiacloprid and thiamethoxam (Table 5). The highest level of resistance was documented to acetamiprid as the most frequently used neonicotinoid in commercial potato fields in Czechia after restriction of thiacloprid and thiamethoxam. Before the restriction by EU regulation, thiacloprid and thiamethoxam were the most frequently used neonicotinoids against CPB in Czechia. Acetamiprid was registered in Czechia in the field-recommended dose of 12 g a.i./ha, while thiacloprid and thiamethoxam were registered in the field-recommended dose of 72 g a.i./ha and 28.8 g a.i./ha, respectively. Hence, the selection pressure on the CPB resistance development to acetamiprid can be higher due to lower application dose.

Similarly, Scott et al. [17] documented different rates of resistance development to various neonicotinoids. First, resistance to acetamiprid was observed, followed by resistance to thiacloprid and thiamethoxam. Similar trends of gradual resistance selection were documented for *Brassicogethes aeneus* and their resistance to class I and II pyrethroids [47].

No cross-resistance between acetamiprid, thiacloprid and thiamethoxam was observed in our experiments, while in other studies, cross-resistance was shown between imidacloprid and thiamethoxam [11]. Grapputo et al. [32] documented different levels of CPB resistance to imidacloprid in one population during the vegetation period. Temporal variations in imidacloprid susceptibility included early time points of susceptibility and later peaks in resistance. Heightened resistance occurred during the second generation and correlated with the increased transcript abundance of multiple mechanisms of resistance, including multiple cuticular protein and cytochrome p450 transcripts [43].

### CPB resistance to insecticides in various regions of Czechia

We tested the hypotheses that CPB populations differ in the level of resistance to insecticides in regions with different climatic conditions and different intensities of CPB control and selection pressure of insecticides. We suppose that the selection of resistance is more intensive in regions with a high intensity of chemical control, i.e., the Central Bohemia and South Moravia regions. The level of CPB resistance to pyrethroids and organophosphates did not differ between regions in Czechia (Table 4). This implies the stability and spread of resistance between the regions. High variability in resistance levels was found in particular regions in Czechia only for acetamiprid. The CPB susceptibilities to the other two neonicotinoids, i.e., thiacloprid and thiamethoxam, were high in all the evaluated regions (Table 4). No differences were found in the levels of resistance to particular neonicotinoids between the evaluated regions of Czechia. CPBs are bivoltine in these regions [9]. Resistant individuals occur first in one region and then spread to neighbouring regions. Several observations indicate that CPBs are weak fliers, but they can engage in long-distance migration in the presence of strong winds [48,49]. The studied regions in Czechia are small in comparison to the USA [32]. This can explain the small regional variability of CPB phenotypes in Czechia.

CPBs evolve insecticide resistance repeatedly across agricultural regions, leveraging similar genetic pathways but different genes, demonstrating a polygenic trait architecture for insecticide resistance that can evolve from standing genetic variation. Despite expectations, Pelissie et al. [30] did not find any support for strong selection on novel mutations or rapid evolution from selection on regulatory genes.

### Antiresistance strategy

Currently, 36 commercial formulations based on pyrethroids, neonicotinoids (acetamiprid), diamides, azadirachtin and spinosad are registered in Czechia for the control of CPBs on potatoes. In addition to chemical insecticides, several biological (*Steinernema carpocapsae*) and physical (polyolefins) means of control are also registered. Resistance to pyrethroids and organophosphates was documented in Czechia in 2004 [9], and very low efficacy against CPBs was shown in our experiments (Tables 3, 4 and S1). Until 2022, no resistance of CPBs to diamides, azadirachtin and spinosad was detected in Czechia. After the restriction of using chlorpyrifos, thiacloprid and thiamethoxam by EU regulation [39–41], insecticides based on diamides, azadirachtin and spinosad are currently the only effective chemical insecticides to control CPBs in Czechia.

The insecticides based on diamides are currently the basis for CPB control in conventional potato growing. The high sensitivity of CPB populations and the stable efficacy of chlorantraniliprole from the diamide group were shown in our experiments (Tables 3 and 6). The insecticides based on azadirachtin showed high variability in efficacy between years (Table 6); due to this variability, they are not suitable for CPB control in intensive potato-growing systems. Rather, they are recommended for CPB control in organic farming for rotation with insecticides based on spinosad and other biological and physical means of control. Insecticides based on spinosad are currently an important part of CPB control in conventional potato growing and organic farming. Spinosad shown high and stable efficacy in our small plot experiments (Table 6). Regarding the risk of selection of resistance to spinosad in CPBs [8,50], it is necessary to rotate spinosad with diamides and azadirachtin in conventional and organic farming applications, respectively.

Based on the results of resistance monitoring, it is not recommended to use pyrethroids and apply acetamiprid only in locations with the preserved high efficacy. The CPB control in the conventional potato growing should be based mainly on diamides and spinosad. However, using of these two groups for sustainable antiresistance strategy is insufficient. Antiresistance strategies in the control of CPBs should comprise careful monitoring of CPB resistance to the insecticides used, the extension of the spectrum of insecticides registered to control CPBs in Czechia and the determination of baseline susceptibility to newly registered insecticides.

## Supporting information

S1 Table List of regions and localities of *Leptinotarsa decemlineata* collection in Czechia in 2017–2022 with their coordinates, date of collection and altitude data. Regions are presented with mean sum of effective temperature (SET) above 10° C from 1st January to 30st November in 2017–2022 and mean temperatures from 1st January to 30st November in 2017–2022.
(PDF)

S2 Table Composite log-dose probit mortality of *Leptinotarsa decemlineata* collected from different regions of Czechia following exposure to lambda-cyhalothrin obtained from the bioassays: Lethal dose for 20 and 50% of the larvae (LC20, LC50; mg/L) and corresponding 95% confidence limits (95% CL; mg/L) and regression slopes with standard error (SE), nd–fit with unreal data (i.e.>999.999 mg/L). (PDF)

S3 Table Composite log-dose probit mortality of *Leptinotarsa decemlineata* collected from different regions of Czechia following exposure to chlorpyrifos obtained from the bioassays: Lethal dose for 50 and 90% of the larvae (LC50, LC90; mg/L) and corresponding 95% confidence limits (95% CL; mg/L) and regression slopes with standard error (SE), nd–fit with unreal data (i.e.>999.999 mg/L). (PDF)

S4 Table Composite log-dose probit mortality of Leptinotarsa decemlineata collected from different regions of Czechia following exposure to acetamiprid obtained from the bioassays: Lethal dose for 50 and 90% of the larvae (LC50, LC90; mg/L) and corresponding 95% confidence limits (95% CL; mg/L) and regression slopes with standard error (SE), nd–fit with unreal data (i.e.>999.999 mg/L), HM–high mortality (i.e.˃95%) at all the evaluated application rates. (PDF)

S5 Table Composite log-dose probit mortality of Leptinotarsa decemlineata collected from different regions of Czechia following exposure to thiacloprid obtained from the bioassays: Lethal dose for 50 and 90% of the larvae (LC50, LC90; mg/L) and corresponding 95% confidence limits (95% CL; mg/L) and regression slopes with standard error (SE), nd–fit with unreal data (i.e.>999.999 mg/L), HM–high mortality (i.e.˃93%) at all the evaluated application rates. (PDF)

S6 Table Composite log-dose probit mortality of Leptinotarsa decemlineata collected from different regions of Czechia following exposure to thiamethoxam obtained from the bioassays: Lethal dose for 50 and 90% of the larvae (LC50, LC90; mg/L) and corresponding 95% confidence limits (95% CL; mg/L) and regression slopes with standard error (SE), nd–fit with unreal data (i.e.>999.999 mg/L). (PDF)

S7 Table Composite log-dose probit mortality of Leptinotarsa decemlineata collected from different regions of Czechia following exposure to chlorantraniliprole obtained from the bioassays: Lethal dose for 50 and 90% of the larvae (LC50, LC90; mg/L) and corresponding 95% confidence limits (95% CL; mg/L) and regression slopes with standard error (SE), nd–fit with unreal data (i.e.>999.999 mg/L), HM–high mortality (i.e.˃95%) at all the evaluated application rates. (PDF)
