# Supplementary material for: Six-year monitoring of pesticide resistance in the Colorado potato beetle (Leptinotarsa decemlineata Say) during a neonicotinoid restriction period
Source: PLoS One. 2024 May 6;19(5):e0303238. doi: 10.1371/journal.pone.0303238 (PMC11073731; doi:10.1371/journal.pone.0303238)
Supplement: S1 Table — Regions are presented with mean sum of effective temperature (SET) above 10° C from 1st January to 30st November in 2017–2022 and mean temperatures from 1st January to 30st November in 2017–2022. (PDF) [file pone.0303238.s001.pdf]

S1 Table. List of regions and localities of *Leptinotarsa decemlineata* collection in Czechia in 2017–2022 with their coordinates, date of collection and altitude data. Regions are presented with mean sum of effective temperature (SET) above 10 °C from 1st January to 30st November in 2017 – 2022 and mean temperatures from 1st January to 30st November in 2017-2022.

Locality	District	GPS	Date of collection	Altitude (m. a. s. l)
**South Moravia mean SET 1402 °C, mean temperature 13.74 °C**				
Popovice 2018	Brno-venkov	49.1041883N, 16.6123308E	19th June	189
Bozice 2018	Znojmo	48.8534033N, 16.2960956E	5th June	198
Zabcice 2022	Brno-venkov	49.0061308N, 16.5825875E	15th June	207
Troubsko 2017	Brno-venkov	49.1737825N, 16.4943606E	4th July	281
Troubsko 2019	Brno-venkov	49.1832997N, 16.4930408E	11th June	305
**Central Bohemia mean SET 1313 °C, mean temperature 13.29 °C**				
Travcice 2021	Litomerice	50.5120767N, 14.1821950E	17th June	147
Travcice 2020	Litomerice	50.5108997N, 14.1870767E	19th May	148
Travcice 2022	Litomerice	50.5127419N, 14.1778175E	25th May	148
Travcice 2018	Litomerice	50.5108089N, 14.1838542E	22nd May	149
Travcice 2017	Litomerice	50.5061208N, 14.1862053E	9th June	150
Hrdly 2019	Litomerice	50.4826275N, 14.1701122E	26th June	159
Obristvi 2018	Melnik	50.3130803N, 14.4658253E	29th May	160
Dolanky nad Ohri 2017	Litomerice	50.4712658N, 14.1849389E	26th June	161
Obristvi 2022	Melnik	50.2881928N, 14.4810025E	30th May	163
Obristvi 2020	Melnik	50.2857661N, 14.4362203E	21st May	167
Obristvi 2021	Melnik	50.2857661N, 14.4362203E	10th June	167
Zalezlice 2019	Melnik	50.2929189N, 14.4293183E	14th June	169
Semice 2017	Nymburk	50.1578033N, 14.8802989E	12th June	179

Semice 2018	Nymburk	50.1585444N, 14.8802672E	30th May	179
Semice 2022	Nymburk	50.1620244N, 14.8585300E	30th May	182
Prerov nad Labem 2018	Nymburk	50.1563961N, 14.8296819E	8th June	184
Stary Vestec 2020	Nymburk	50.1470597N, 14.8449903E	19th May	187
Semice 2021	Nymburk	50.1584469N, 14.8688297E	7th June	195
Libocany 2019	Louny	50.3481586N, 13.5044953E	11th June	212
Celakovice 2018	Prague-east	50.1485278N, 14.7256389E	28th May	216
Podsedice 2022	Litomerice	50.4548331N, 13.9566939E	2nd June	253
Holany 2022	Ceska Lipa	50.618158N, 14.502757E	13th June	265
Ruzyne 2022	Prague	50.0872672N, 14.3003456E	10th June	347
Ruzyne 2017	Prague	50.0877775N, 14.2997917E	6th June	351
Ruzyne 2018	Prague	50.0877775N, 14.2997917E	12th June	351
Ruzyne 2020	Prague	50.0877111N, 14.3000558E	18th May	351
Ruzyne 2021	Prague	50.0880153N, 14.2987417E	22nd June	355
Ruzyne 2019	Prague	50.0877775N, 14.2997917E	17th July	356
**Bohemian-Moravian Highlands**				
**mean SET 1214 °C, mean temperature 12.79 °C**				
Svitavy 2020	Svitavy	49.7511406N, 16.4802672E	15th July	443
Svitavy 2021	Svitavy	49.7511292N, 16.4817153E	20th July	443
Svitavy 2022	Svitavy	49.7511406N, 16.4802672E	15th June	443
Vilemov 2017	Prostejov	49.6419444N, 16.9861111E	7th July	446
Valecov 2019	Havlickuv Brod	49.6476294N, 15.4946192E	27th June	469
Valecov 2022	Havlickuv Brod	49.6476333N, 15.4946167E	15th June	470
Zahori u Milicina 2022	Benesov	49.5639181N, 14.6791581E	17th June	550
Utechovicky u Pelhrimova 2017	Pelhrimov	49.4510781N, 15.0999564E	21st July	591
**Central and North Moravia**				
**mean SET 1171 °C, mean temperature 12.65 °C**				
Javornik 2018	Jesenik	50.3915278N, 17.0105278E	22nd June	283
Frycovice 2021	Frydek-Mistek	49.6615917N, 18.2373667E	13th July	311

Dolni Zivotice 2018	Opava	49.9053603N, 17.7985611E	31st May	327
Vicov 2018	Prostejov	49.4912747N, 16.9693658E	23th July	335
Vrsovice 2021	Opava	49.8817075N, 17.9347800E	28th July	348
Vrsovice 2020	Opava	49.8817075N, 17.9347800E	10th July	373
Vrsovice 2019	Opava	49.8815842N, 17.9347467E	20th June	375
**South and West Bohemia**				
**mean SET 1158 °C, mean temperature 12.56 °C**				
Stankov 2019	Domazlice	49.5510433N, 13.0563383E	2nd July	375
Chotikov 2022	Plzen-north	49.788056N, 13.317778E	13th June	383
Ostretice 2018	Klatovy	49.4226483N, 13.3367022E	15th June	408
Nemcovice 2021	Rokycany	49.8783986N, 13.5764317E	24th June	408
Nemcovice 2022	Rokycany	49.8769311N, 13.5748839E	14th June	412
Chvalenice 2022	Plzen-town	49.647118N, 13.49515E	13th June	415
Pracejovice 2020	Strakonice	49.2525861N, 13.8701511E	8th July	417
Slavosovice 2021	Klatovy	49.3938458N, 13.3362372E	8th July	417
Novosedly 2021	Strakonice	49.2605361N, 13.7812997E	30th June	421
Ostretice 2022	Klatovy	49.4007808N, 13.3233656E	12th July	426
Tesovice 2017	Domazlice	49.5112294N, 13.1444242E	3rd July	436
Stryckovice 2018	Domazlice	49.5112222N, 13.1444167E	14th June	440
Drachkov 2018	Strakonice	49.2497222N, 13.8319444E	21st June	447
Drachkov 2022	Strakonice	49.2479194N, 13.8484389E	21st June	452
Procevily 2022	Pribram	49.5579961N, 13.9026356E	18th June	443
Procevily 2018	Pribram	49.5527750N, 13.8969694E	13th June	532
Bezdekov pod Tremsinem 2022	Pribram	49.5829092N, 13.8996142E	20th June	521
Vysoka u Pribrame 2017	Pribram	49.5016442N, 13.9006236E	28th June	539
Procevily 2020	Pribram	49.5537167N, 13.8836167E	16th June	572
Procevily 2021	Pribram	49.5572514N, 13.8897181E	24th June	574
Procevily 2019	Pribram	49.5572514N, 13.8897181E	2nd July	576
