# Supplementary material for: Six-year monitoring of pesticide resistance in the Colorado potato beetle (Leptinotarsa decemlineata Say) during a neonicotinoid restriction period
Source: PLoS One. 2024 May 6;19(5):e0303238. doi: 10.1371/journal.pone.0303238 (PMC11073731; doi:10.1371/journal.pone.0303238)
Supplement: S2 Table — (PDF) [file pone.0303238.s002.pdf]

**S2 Table. Composite log-dose probit mortality of *Leptinotarsa decemlineata* collected from different regions of Czechia following exposure to *lambda*-cyhalothrin obtained from the bioassays: lethal dose for 20 and 50% of the larvae ($LC_{20}$, $LC_{50}$; mg/L) and corresponding 95% confidence limits (95% CL; mg/L) and regression slopes with standard error (SE), nd – fit with unreal data (i.e.>999.999 mg/L).**

| year | population | $LC_{20}$ mg/L | 95% CL | $LC_{50}$ mg/L | 95% CL | slope | mortality (%) at recommended application rate | mortality (%) at 281 mg/L rate |
|---|---|---|---|---|---|---|---|---|
| 2017 | Travčice | 2,830 | nd | 30,530 | nd | 0.81±0.84 | 0 | 3.85 |
| | Semice | 166 | 82.0-428 | 698 | 315-21,575 | 1.35±0.46 | 3.33 | 33.3 |
| | Ruzyně | 196 | 66.0-3,578 | 3,633 | 573-nd | 0.66±0.22 | 6.67 | 20.0 |
| | Troubsko | 195 | 101-518 | 633 | 315-32,908 | 1.65±0.63 | 0 | 26.7 |
| | Těšovice | 16.1 | 3.95-34.3 | 103 | 50.5-250 | 1.05±0.23 | 30.0 | 36.7 |
| | Vysoká u Příbramě | 1,005 | nd | 5,848 | nd | 1.10±0.85 | 0 | 6.67 |
| | Vilémov | 328 | 127–nd | 2,833 | 525-nd | 0.90±0.45 | 3.33 | 20.0 |
| | Útěchovičky u Pelhřimova | 27.3 | 0.20-66.0 | 360 | 144-78,155 | 0.75±0.30 | 16.7 | 46.7 |
| 2018 | Travčice | 36.8 | 17.5-58.5 | 136 | 88.0-242 | 1.48±0.28 | 10.3 | 63.3 |
| | Obříství | 50.5 | 27.3-70.8 | 100 | 71.5-133 | 2.83±0.54 | 0 | 86.7 |
| | Přerov nad Labem | 115 | 21.5-155,833 | 7,523 | 603-nd | 0.46±0.21 | 10.9 | 28.7 |
| | Čelákovice | 11.9 | 1.93-26.5 | 103 | 54.3-219 | 1.15±0.27 | 29.6 | 66.7 |
| | Ruzyně | 20.6 | 7.80-36.5 | 118 | 69.0-248 | 1.11±0.22 | 26.7 | 60.0 |
| | Popovice | 14.5 | 5.43-25.8 | 76.8 | 46.3-140 | 1.16±0.21 | 36.7 | 70.0 |
| | Javorník | 22.2 | 4.28-51.8 | 368 | 137-5,435 | 0.69±0.20 | 26.7 | 53.3 |
| | Dolní Životice | 4.83 | 0.53-12.5 | 69.3 | 32.0-208 | 0.73±0.18 | 27.6 | 75.9 |
| | Vícov | 101 | 34.5-215 | 575 | 253-19,018 | 1.11±0.38 | 3.33 | 33.3 |
| | Strýčkovice | 13.3 | 3.90-28.0 | 121 | 61.8-275 | 0.88±0.16 | 33.3 | 57.1 |
| | Pročevily | 14.5 | 5.43-25.8 | 76.8 | 46.3-140 | 1.15±0.21 | 36.7 | 70.0 |
| 2019 | Hrdly | 72.5 | nd | 32,355 | nd | 0.32±0.20 | 10.3 | 27.6 |
| | Zálezlice | 124 | 62.5-265 | 908 | 385-5,530 | 0.97±0.21 | 3.7 | 31.7 |
| | Ruzyně | 270 | nd | 435 | nd | 4.06±279 | 0 | 30.0 |
| | Libočany | 575 | 115-nd | 12,593 | 848-nd | 0.63±0.30 | 0 | 16.7 |
| | Troubsko | 60.5 | 25.8-112 | 241 | 130-625 | 1.40±0.30 | 0 | 56.7 |
| | Pročevily | 13.4 | 3.38-30.3 | 135 | 59.0-585 | 0.84±0.20 | 16.7 | 63.3 |
| | Valečov | 256 | nd | 410 | nd | 4.15±231 | 0 | 25.0 |
| 2020 | Travčice | 43.3 | 2.73-350 | 2,883 | 355-nd | 0.46±0.20 | 20.0 | 42.9 |
| | Obříství | 125 | 62.5-265 | 598 | 278-5,405 | 1.24±0.35 | 6.67 | 40.0 |
| | Starý Vestec | 490 | nd | 21,788 | nd | 0.51±0.27 | 10.0 | 13.3 |
| | Ruzyně | 483 | 172-nd | 3,960 | 655-nd | 0.92±0.42 | 3.30 | 16.7 |
| | Vršovice | 25.5 | 2.95-69.8 | 668 | 185-115,883 | 0.59±0.20 | 26.7 | 34.5 |
| | Pracejovice | 62.5 | 22.6-103 | 233 | 144-560 | 1.56±0.41 | 3.58 | 51.8 |
| | Pročevily | 2.98 | 0.60-6.80 | 21.3 | 10.3-37.5 | 1.05±0.20 | 55.2 | 82.8 |
| | Svitavy | 78.0 | 33.6-171 | 590 | 243-6,078 | 0.96±0.25 | 6.67 | 43.3 |

| | | | | | | | | |
|---|---|---|---|---|---|---|---|---|
| 2021 | Travčice | 230 | 65.0-nd | 7,625 | 700-nd | 0.55±0.24 | 6.70 | 11.1 |
| | Obříství | 43.0 | 0.88-90.5 | 308 | 154-7,108 | 1.15±0.45 | 3.54 | 42.8 |
| | Semice | 194 | nd | 1,268 | nd | 1.32±0.99 | 6,70 | 23.3 |
| | Fryčovice | 95.3 | 46.3-169 | 403 | 215-1,938 | 1.34±0.36 | 3.40 | 39.3 |
| | Vršovice | 380 | 118–nd | 6,383 | 725-nd | 0.69±0.31 | 6.67 | 16.7 |
| | Němčovice | 221 | 88.8-4,400 | 2,248 | 493-nd | 0.84±0.32 | 3.40 | 16.7 |
| | Slavošovice | 105 | 16.0-201 | 490 | 242-22,093 | 1.40±0.55 | 1.75 | 33.3 |
| | Novosedly | 91.0 | 23.5-200 | 595 | 253-17,758 | 1.10±0.38 | 10.4 | 38.0 |
| | Pročevily | 82.0 | 42.8-123 | 241 | 160-493 | 1.80±0.41 | 3.33 | 56.7 |
| | Svitavy | 56.0 | 26.8-90.0 | 220 | 135-500 | 1.42±0.31 | 6.67 | 53.3 |
| 2022 | Travčice | 290 | nd | 355 | nd | 9.60±564 | 0 | 16.7 |
| | Obříství | 1,615 | nd | 12,223 | nd | 1.02±1.14 | 0 | 6.67 |
| | Semice | 335 | 96-nd | 7,688 | 735-nd | 0.62±0.28 | 0 | 14.3 |
| | Holany | 103 | 41.5-335 | 1,058 | 328-61,145 | 0.83±0.26 | 3.33 | 30.0 |
| | Ruzyně | 5.18 | 0.001–22.4 | 598 | 119-nd | 0.41±0.18 | 40.0 | 48.3 |
| | Žabčice | 3.90 | 0.001–17.1 | 373 | 91.5-nd | 0.43±0.18 | 30.0 | 50.0 |
| | Němčovice | 146,998 | nd | nd | nd | 0.32±0.39 | 3.33 | 3.33 |
| | Chvalenice | 27.5 | 7.65-56.8 | 290 | 130-1,863 | 0.82±0.21 | 26.7 | 43.3 |
| | Ostřetice | 172 | 68.0-1,605 | 1,994 | 455-nd | 0.79±0.28 | 10.0 | 26.7 |
| | Drachkov | 310 | 162-110,055 | 1,130 | 410-nd | 1.50±0.69 | 0 | 17.2 |
| | Pročevily | 58.5 | 18.4-163 | 863 | 260-46,438 | 0.72±0.22 | 6.67 | 36.7 |
| | Bezděkov pod Třemšínem | 360 | 158-48,818 | 1,968 | 508-nd | 1.14±0.48 | 3.20 | 21.4 |
| | Svitavy | 88.8 | 42.0-168 | 455 | 225-2,656 | 1.19±0.31 | 6.67 | 40.0 |
| | Valečov | 99.8 | 50.5-175 | 410 | 223-1,763 | 1.37±0.35 | 6.67 | 46.7 |
| | Záhoří u Miličína | 18.0 | 5.65-34.5 | 140 | 75.3-383 | 0.94±0,20 | 25.8 | 56.7 |
