# Supplementary material for: Six-year monitoring of pesticide resistance in the Colorado potato beetle (Leptinotarsa decemlineata Say) during a neonicotinoid restriction period
Source: PLoS One. 2024 May 6;19(5):e0303238. doi: 10.1371/journal.pone.0303238 (PMC11073731; doi:10.1371/journal.pone.0303238)
Supplement: S3 Table — (PDF) [file pone.0303238.s003.pdf]

S3 Table. Composite log-dose probit mortality of *Leptinotarsa decemlineata* collected from different regions of Czechia following exposure to chlorpyrifos obtained from the bioassays: lethal dose for 50 and 90% of the larvae (LC_{50}, LC_{90}; mg/L) and corresponding 95% confidence limits (95% CL; mg/L) and regression slopes with standard error (SE), nd – fit with unreal data (i.e.>999.999 mg/L).

year	population	LC_{50} mg/L	95% CL	LC_{90} mg/L	95% CL	slope	mortality (%) in recommended application rate
2017	Travčice	1,200	468-8,650	235,200	21,588-nd	0.56±0.14	30.0
	Dolánky nad Ohří	12,563	nd	19,375	nd	6.81±309	0
	Semice	1,888	940-7,123	46,495	10,605-nd	0.92±0.20	20.0
	Ruzyně	1,475	950-3,068	10,550	4,468-69,633	1.50±0.30	34.5
	Troubsko	928	565-1,605	8,160	4,070-24,058	1.36±0.18	33.3
	Vysoká u Příbramě	9,380	5,445-21,858	89,623	33,905-594,385	1.31±0.23	6.67
	Vilémov	4,073	2,228-9,670	88,360	28,490-nd	0.96±0.15	16.7
	Útěchovičky u Pelhřimova	398	241-640	3,483	1,910-8,673	1.36±0.19	46.7
2018	Travčice	5,100	3,240-9,483	38,035	17,578-155,230	1.47±0.24	10.0
	Přerov nad Labem	8,555	1,125-12,403	22,838	15,540-240,380	3.09±1.27	2.53
	Čelákovice	3,488	2,050-5,328	10,035	6,423-22,920	2.96±0.66	6.80
	Ruzyně	1,333	978-1,923	5,168	3,198-12,428	2.17±0.37	30.0
	Javorník	1,118	793-1,595	5,045	3,118-11,880	1.96±0.33	56.7
	Dolní Životice	863	165-2,340	150,305	20,713-nd	0.57±0.19	51.7
	Vícov	2,330	1,673-3,543	8,620	5,154-148,544	2.26±0.42	23.3
	Ostřetice	1,708	1,148-2,640	11,260	6,165-32,150	1.56±0.25	36.7
	Strýčkovice	3,368	2,450-4,983	9,623	5,215-20,388	2.81±0.46	4.00
	Pročevily	950	690-1,330	3,763	2,383-8,838	2.15±0.38	53.3
2019	Zálezlice	1,224	685-2,243	12,280	5,565-53,665	1.28±0.23	64.3
	Libočany	2,636	1,590-4,708	16,648	8,378-52,773	1.60±0.25	27.6
	Ruzyně	3,659	1,985-7,460	19,533	9,173-77,833	1.76±0.33	15.0
	Troubsko	8,614	5,033-15,660	44,010	22,345-165,540	1.81±0.36	3.30
	Vršovice	2,548	1,320-5,848	44,955	15,058-435,378	1.03±0.20	36.7
	Staňkov	5,435	3,215-8,840	20,740	12,278-47,850	2.20±0.38	3.30
	Pročevily	500	161-1,018	3,343	1,455-89,340	1.55±0.52	55.0
	Valečov	7,174	nd	12,010	nd	5.73±234	0
2020	Travčice	1,042	723-1,523	4,483	2,735-11,020	2.02±0.36	60.0
	Obříství	925	620-1,353	4,910	2,990-11,123	1.77±0.27	43.3
	Vršovice	798	600-1,090	2,108	1,460-4,013	3.04±0.53	53.6
	Pracejovice	32,762	14,594-96,918	955,628	249,220-nd	1.83±0.37	0.14
	Pročevily	1,364	938-1,968	4,590	2,945-10,628	2.47±0.48	24.2
