# Supplementary material for: Six-year monitoring of pesticide resistance in the Colorado potato beetle (Leptinotarsa decemlineata Say) during a neonicotinoid restriction period
Source: PLoS One. 2024 May 6;19(5):e0303238. doi: 10.1371/journal.pone.0303238 (PMC11073731; doi:10.1371/journal.pone.0303238)
Supplement: S4 Table — (PDF) [file pone.0303238.s004.pdf]

S4 Table. Composite log-dose probit mortality of *Leptinotarsa decemlineata* collected from different regions of Czechia following exposure to acetamiprid obtained from the bioassays: lethal dose for 50 and 90% of the larvae (LC_{50}, LC_{90}; mg/L) and corresponding 95% confidence limits (95% CL; mg/L) and regression slopes with standard error (SE), nd – fit with unreal data (i.e.>999.999 mg/L), HM – high mortality (i.e.>95%) at all the evaluated application rates.

year	population	LC_{50} mg/L	95% CL	LC_{90} mg/L	95% CL	slope	mortality (%) in recommended application rate
2017	Travčice	35.5	14.7-85.5	1,695	358-nd	0.76±0.24	46.7
	Semice	104	68.5-213	773	323-6,285	1.47±0.33	26.7
	Dolánky nad Ohří	318	223-490	1,180	713-490	2.32±0.80	3.33
	Ruzyně	31.5	18.0-74.3	560	177-7,655	1.03±0.22	40.0
	Troubsko	194	134-313	1,013	555-2,790	1.79±0.26	7.41
	Těšovice	42.5	33.3-52.0	95.0	72.8-164	3.67±0.77	33.3
	Vilémov	29.0	19.5-41.3	169	104-385	1.68±0.27	50.0
	Útěchovičky u Pelhřimova	11.2	7.70-15.3	35.3	25.1-58.8	2.56±0.39	86.7
2018	Travčice	22.4	14.1-35.5	180	98.0-468	1.42±0.20	69.0
	Obříství	16.8	6.18-33.0	440	185-2,320	0.90±0.18	73.3
	Přerov nad Labem	13.4	8.33-21.2	128	68.8-345	1.31±0.19	64.9
	Popovice	13.8	7.68-24.2	248	112-898	1.02±0.15	46.7
	Čelákovice	156	565-443	9,955	2,030-nd	0.75±0.19	18.5
	Javorník	35.3	18.6-66.5	1,030	413-4,595	0.87±0.13	73.3
	Ruzyně	79.8	53.0-123	468	265-1,208	1.67±0.25	36.7
	Dolní Životice	36.8	19.4-71.8	1,130	425-5,738	0.86±0.13	34.5
	Vícov	41.8	19.8-109	1,105	280-84,985	0.90±0.25	50.0
	Ostřetice	84.5	44.8-183	2,765	925-17,498	0.85±0.12	20.0
	Strýčkovice	67.5	38.0-126	1,403	583-5,778	0.97±0.14	46.7
	Drachkov	74.8	48.0-123	535	530-1,530	1.50±0.22	30.0
	Pročevily	19.1	13.1-27.8	77.3	48.5-166	2.11±0.33	56.7
2019	Hrdly	18.3	13.5-24.8	46.8	33.0-86.5	3.15±0.57	74.2
	Zálezlice	60.0	41.8-90.8	233	141-583	2.17±0.38	20.0
	Libočany	22.6	10.8-41.0	315	130-2,590	1.12±0.26	75.9
	Ruzyně	10.3	5.78-16.0	36.5	22.0-117	2.33±0.58	85.0
	Troubsko	67.5	45.0-111	338	181-1,178	1.84±0.36	33.3
	Vršovice	73.3	40.3-196	1,100	330-30,853	1.09±0.28	53.3
	Staňkov	58.3	38.0-95.8	328	172-1,190	1.71±0.33	43.3
	Pročevily	20.1	nd	26.5	nd	10.6±399	96.7
	Valečov	58.3	44.8-78.3	113	83.0-188	4.46±0.77	10.0
2020	Travčice	713	nd	nd	nd	0.19±0.24	36.7
	Obříství	29.5	18.6-47.0	189	101-618	1.59±0.29	43.3

	Ruzyně	27.3	13.0-54.3	485	168-8,343	1.02±0.26	46.7
	Pracejovice	51.3	33.8-76.3	202	123-535	2.18±0.44	31.1
2021	Travčice	17.6	6.63-32.5	250	106-2,210	1.18±0.30	59.3
	Obříství	9.60	5.75-13.9	34.0	22.2-78.8	2.42±0.52	85.7
	Semice	10.9	4.33-18.7	97.3	51.5-373	1.43±0.33	70.4
	Ruzyně	1,668	nd	511,173	nd	0.52±0.28	10.7
	Fryčovice	53.0	16.9-1,010	7,338	578-nd	0.60±0.24	48.3
	Vršovice	HM					100
	Němčovice	108	70.8-199	498	250-2,205	1.93±0.40	6.70
	Pročevily	94.3	59.5-189	600	268-3,645	1.59±0.34	20.0
2022	Travčice	136	103-154	209	186-263	6.88±1.68	86.7
	Obříství	7.30	3.90-10.8	28.8	18.4-73.5	2.19±0.51	89.7
	Semice	13.2	2.48-29.3	558	147-101,333	0.79±0.25	53.3
	Podsedice	31.5	nd	45.0	nd	8.34±277	42.9
	Holany	40.8	14.4-188	3,005	410-nd	0.69±0.24	50.0
	Ruzyně	137	62.3-1,093	3,263	575-nd	0.93±0.27	20.0
	Žabčice	31.8	22.8-45.0	97.8	64.5-201	2.62±0.44	36.7
	Chotíkov	0.53	0.001–0.86	32.8	7.78-nd	0.71±0.35	88.9
	Němčovice	173	90.0-768	1,875	510-89,673	1.24±0,33	17.9
	Drachkov	12.5	5.95-21.0	118	58.8-555	1.32±0,30	50.0
	Pročevily	4.98	1.73-7.90	22.2	13.9-75.0	2.01±0.55	93.3
	Bezděkov pod Třemšínem	37.3	20.5-74.8	483	180-5,470	1.15±0.27	40.0
	Svitavy	2.20	0.07-5.68	30.5	14.9-166	1.13±0.36	93.3
	Záhoří u Miličína	10.7	3.88-19.3	141	66.3-798	1.14±0.27	86.7
