# Supplementary material for: Six-year monitoring of pesticide resistance in the Colorado potato beetle (Leptinotarsa decemlineata Say) during a neonicotinoid restriction period
Source: PLoS One. 2024 May 6;19(5):e0303238. doi: 10.1371/journal.pone.0303238 (PMC11073731; doi:10.1371/journal.pone.0303238)
Supplement: S5 Table — (PDF) [file pone.0303238.s005.pdf]

S5 Table. Composite log-dose probit mortality of *Leptinotarsa decemlineata* collected from different regions of Czechia following exposure to thiacloprid obtained from the bioassays: lethal dose for 50 and 90% of the larvae (LC_{50}, LC_{90}; mg/L) and corresponding 95% confidence limits (95% CL; mg/L) and regression slopes with standard error (SE), nd – fit with unreal data (i.e.>999.999 mg/L), HM – high mortality (i.e.>93%) at all the evaluated application rates.

year	population	LC_{50} mg/L	95% CL	LC_{90} mg/L	95% CL	slope	mortality (%) in recommended application rate
2017	Travčice	273	219-333	595	463-940	3.80±0.7	23.3
	Dolánky nad Ohří	295	229-373	790	583-1,363	3.01±0.53	23.3
	Semice	518	398-728	1,643	1,058-3,825	2.55±0.46	20.0
	Ruzyně	78.5	52.0-115	548	338-1,125	1.52±0.20	73.3
	Troubsko	308	120-510	2,692	1,105-nd	1.36±0.50	35.7
	Těšovice	120	73.8-190	688	388-1,813	1.68±0.28	46.7
	Vysoká u Příbramě	238	185-305	705	510-1,213	2.72±0.42	33.3
	Vilémov	88.0	55.5-124	418	280-795	1.89±0.31	76.7
	Útěchovičky u Pelhřimova	53.8	34.5-71.5	142	104-253	3.06±0.66	93.3
2018	Travčice	38.5	nd	54.5	nd	8.50±557	100
	Obříství	93.5	57.0-158	1,033	478-4,420	1.23±0.22	60.0
	Semice	35.0	22.0-52.5	228	137-510	1.58±0.24	83.9
	Přerov nad Labem	162.25	nd	194.25	nd	16.2±1,013	75.4
	Čelákovice	48.8	24.7-79.0	440	240-1,350	1.43±0,21	81.5
	Ruzyně	97.5	70.0-129	288	209-468	2.73±0.42	83.3
	Popovice	37.8	nd	54.0	nd	8.24±526	100
	Božice	50.8	31.0-78.5	428	237-1,153	1.38±0.22	83.3
	Javorník	106	66.3-177	1,045	500-4,055	1.29±0.22	73.3
	Dolní Životice	72.3	41.8-126	1,018	440-,5300	1.12±0.21	66.7
	Vícov	88.0	55.5-124	418	280-795	1.76±0.37	60.0
	Ostřetice	168	114-265	985	525-3,330	1.67±0.30	35.7
	Strýčkovice	117	78.0-174	698	403-1,845	1.65±0.28	76.7
	Drachkov	168	nd	200	nd	16.8±1,012	70.0
	Pročevily	26.3	19.3-35.3	63.3	45.0-119	3.35±0.65	100
2019	Hrdly	HM					93.1
	Zálezlice	52.3	33.8-72.8	173	120-313	2.48±0.45	93.3
	Libočany	345	238-nd	680	415-nd	4.34±1.30	11.1
	Ruzyně	71.3	47.0-101	192	132-370	2.97±0.48	85.0
	Troubsko	87.0	43.0-140	755	373-4,720	1.37±0.34	66.7
	Vršovice	70.8	42.5-102	333	212-788	1.91±0.38	63.3
	Staňkov	53.5	nd	70.8	nd	10.6±116	100
	Pročevily	81.0	nd	96.8	nd	16.5±1,042	100

Valečov	36.0	12.1-59.8	238	143-723	1.57±0.39	66.7
