# Supplementary material for: Six-year monitoring of pesticide resistance in the Colorado potato beetle (Leptinotarsa decemlineata Say) during a neonicotinoid restriction period
Source: PLoS One. 2024 May 6;19(5):e0303238. doi: 10.1371/journal.pone.0303238 (PMC11073731; doi:10.1371/journal.pone.0303238)
Supplement: S6 Table — (PDF) [file pone.0303238.s006.pdf]

S6 Table. Composite log-dose probit mortality of *Leptinotarsa decemlineata* collected from different regions of Czechia following exposure to thiamethoxam obtained from the bioassays: lethal dose for 50 and 90% of the larvae (LC_{50}, LC_{90}; mg/L) and corresponding 95% confidence limits (95% CL; mg/L) and regression slopes with standard error (SE), nd – fit with unreal data (i.e.>999.999 mg/L).

year	population	LC_{50} mg/L	95% CL	LC_{90} mg/L	95% CL	slope	mortality (%) in recommended application rate
2017	Travčice	7.85	5.68-10.9	22.8	15.6-43.5	2.77±0.47	100
	Dolánky nad Ohří	320.	22.9-40.8	70.3	54.8-99.5	3.75±0.63	90.0
	Ruzyně	10.2	6.90-14.7	48.3	31.0-94.3	1.90±0.27	90.0
2018	Travčice	10.7	7.85-14.6	27.8	19.3-52.0	3.08±0.54	100
	Obříství	2.60	nd	3.53	nd	9.73±568	100
	Přerov nad Labem	9.78	nd	13.9	nd	8.33±555	100
	Čelákovice	1.13	0.005-4.10	179	49.8-28,330	0.62±0.21	85.2
	Ruzyně	13.5	nd	18.2	nd	9.90±494	100
	Javorník	15.4	10.2-22.1	79.5	50.8-160	1.80±0.26	80.0
	Dolní Životice	6.33	3.50-9.95	52.8	30.5-127	1.39±0.22	96.8
	Vícov	12.2	8.15-18.5	59.3	34.5-154	1.86±0.32	83.9
	Ostřetice	21.3	15.4-28.3	60.8	43.8-101	2.81±0.43	79.3
	Strýčkovice	14.5	10.6-19.4	50.0	35.0-87.0	2.38±0.34	83.3
	Drachkov	14.8	10.2-21.1	67.0	43.8-127	1.96±0.27	90.0
	Pročevily	5.73	4.25-7.48	12.0	8.98-19.2	4.01±0.74	100
