# Supplementary material for: Six-year monitoring of pesticide resistance in the Colorado potato beetle (Leptinotarsa decemlineata Say) during a neonicotinoid restriction period
Source: PLoS One. 2024 May 6;19(5):e0303238. doi: 10.1371/journal.pone.0303238 (PMC11073731; doi:10.1371/journal.pone.0303238)
Supplement: S7 Table — (PDF) [file pone.0303238.s007.pdf]

S7 Table. Composite log-dose probit mortality of *Leptinotarsa decemlineata* collected from different regions of Czechia following exposure to chlorantraniliprole obtained from the bioassays: lethal dose for 50 and 90% of the larvae (LC_{50}, LC_{90}; mg/L) and corresponding 95% confidence limits (95% CL; mg/L) and regression slopes with standard error (SE), nd – fit with unreal data (i.e.>999.999 mg/L), HM – high mortality (i.e.>95%) at all the evaluated application rates.

year	population	LC_{50} mg/L	95% CL	LC_{90} mg/L	95% CL	slope	mortality (%) in recommended application rate
2020	Prague	0.13	0.08-0.27	0.63	0.31-2.02	1.95±0.34	96.7
	Travčice	0.22	0.11-0.42	1.54	0.73-5.22	1.50±0.26	86.7
	Obříství	0.14	0.07-0.27	0.87	0.42-3.07	1.61±0.30	93.3
2021	Prague	0.06	nd	0.12	nd	4.31±206	100
	Travčice	HM					95.0
	Pročevily	0.15	0.08-0.28	0.88	0.53-2.97	1.63±0.28	93.3
	Semice	HM					100
	Obříství	0.04	nd	0.08	nd	3.75±225	100
	Fryčovice	0.17	0.09-0.32	0.92	0.45-2.92	1.73±0.29	93.1
2022	Prague	0.05	nd	0.11	nd	4.06±163	100
	Travčice	HM					100
	Semice	0.04	nd	0.09	nd	3.84±172	100
	Obříství	0.07	nd	0.13	nd	4.39±162	100
	Němčovice	0.05	nd	0.10	nd	3.90±170	100
	Drachkov	0.06	nd	0.11	nd	4.14±160	100
	Svitavy	0.04	nd	0.09	nd	3.84±171	100
	Žabčice	0.04	nd	0.09	nd	3.80±173	100
